# Serum 25-Hydroxyvitamin D and Risk of Lung Cancer in Male Smokers: A Nested Case-Control Study

**DOI:** 10.1371/journal.pone.0020796

**Published:** 2011-06-10

**Authors:** Stephanie J. Weinstein, Kai Yu, Ronald L. Horst, Dominick Parisi, Jarmo Virtamo, Demetrius Albanes

**Affiliations:** 1 Division of Cancer Epidemiology and Genetics, National Cancer Institute, National Institutes of Health, Bethesda, Maryland, United States of America; 2 Heartland Assays, Inc., Ames, Iowa, United States of America; 3 Information Management Services, Inc., Silver Spring, Maryland, United States of America; 4 Department of Chronic Disease Prevention, National Institute for Health and Welfare, Helsinki, Finland; Harvard Medical School, United States of America

## Abstract

**Background:**

A role for vitamin D in cancer risk reduction has been hypothesized, but few data exist for lung cancer. We investigated the relationship between vitamin D status, using circulating 25-hydroxyvitamin D [25(OH)D], and lung cancer risk in a nested case-control study within the Alpha-Tocopherol, Beta-Carotene Cancer Prevention Study of Finnish male smokers.

**Methods:**

Lung cancer cases (n = 500) were randomly selected based on month of blood collection, and 500 controls were matched to them based on age and blood collection date. Odds ratios (ORs) and 95% confidence intervals (CIs) were calculated using multivariate-adjusted conditional logistic regression. To account for seasonal variation in 25(OH)D concentrations, season-specific and season-standardized quintiles of 25(OH)D were examined, and models were also stratified on season of blood collection (darker season = November–April and sunnier season = May–October). Pre-determined, clinically-defined cutpoints for 25(OH)D and 25(OH)D as a continuous measure were also examined.

**Results:**

Overall, 25(OH)D was not associated with lung cancer. Risks were 1.08 (95% CI 0.67–1.75) and 0.83 (95% CI 0.53–1.31) in the highest vs. lowest season-specific and season-standardized quintiles of 25(OH)D, respectively, and 0.91 (95% CI 0.48–1.72) for the ≥75 vs. <25 nmol/L clinical categories. Inverse associations were, however, suggested for subjects with blood collections from November–April, with ORs of 0.77 (95% CI 0.41–1.45, p-trend = 0.05) and 0.65 (95% CI 0.37–1.14, p-trend = 0.07) in the highest vs. lowest season-specific and season-standardized quintiles of 25(OH)D, respectively, and 0.61 (95% CI 0.24–1.52, p-trend = 0.01) for ≥75 vs. <25 nmol/L. We also found 11% lower risk for a 10 nmol/L increase in 25(OH)D in the darker season based on the continuous measure (OR = 0.89, 95% CI 0.81–0.98, p = 0.02).

**Conclusion:**

In this prospective study of male smokers, circulating 25(OH)D was not associated with lung cancer risk overall, although inverse associations were suggested among those whose blood was drawn during darker months.

## Introduction

Higher vitamin D intake or status has been hypothesized to be associated with reduced risk of several cancers, including colorectal, breast, and prostate [1], [2]; however, few studies have examined the association between vitamin D status and risk of lung cancer. Circulating 25-hydroxyvitamin D [25(OH)D], the accepted biomarker of vitamin D status, was not associated with lung cancer mortality in an analysis of the National Health and Nutrition Examination Survey (NHANES) [3], but in a recent reanalysis of the NHANES data, incorporating more cases and longer follow-up, higher 25(OH)D was significantly associated with increased lung cancer mortality in men [4]. Serum 25(OH)D was not associated with lung cancer incidence overall in a Finnish cohort study [5], although inverse associations were observed among women and those younger than 50 years [5].

In order to examine the association between circulating vitamin D and lung cancer risk, we conducted a nested case-control study within the Alpha-Tocopherol, Beta-Carotene Cancer Prevention (ATBC) Study cohort of male smokers in Finland (latitude of study area 60–64°N) with 500 lung cancer cases and up to 20 years of follow-up. Multiple approaches were used to address seasonal variation in 25(OH)D concentrations.

## Methods

### Ethics statement

The study was approved by the institutional review boards of the U.S. National Cancer Institute and the National Public Health Institute of Finland, with written informed consent obtained from each participant.

### Study population

The overall design, rationale, and objectives of the ATBC Study have been published [6].

The Study was a randomized, double-blind, placebo-controlled, primary prevention trial with daily supplementation of α-tocopherol (50 mg/day), β-carotene (20 mg/day), both, or placebo. Participants (n = 29,133) aged 50–69 years, who smoked at least five cigarettes per day, were recruited from southwestern Finland from 1985–1988. Study supplementation continued for 5–8 years (median 6.1 years) until death or trial closure (April 30, 1993).

### Case identification and control selection

Incident lung cancer cases (n = 500) were randomly selected from among 2,948 eligible cases (International Classification of Diseases 9, code 162) diagnosed through April 30, 2005. The cases were identified through the Finnish Cancer Registry, which provides nearly 100% case ascertainment [7]. Based on month of baseline blood collection, 50 cases were randomly selected from each month, with 50 cases total selected from June–August since there were few summer blood collections. For cases diagnosed through April 1999 (n = 318), one or two study physicians reviewed medical records for diagnostic confirmation and staging. Cases diagnosed since May 1999 (n = 182) had only the Finnish Cancer Registry data for site, histology, and date of diagnosis. Histology data were available for all but 87 cases. The main histologic subtypes were small cell carcinoma (n = 100), squamous cell carcinoma (n = 179), and adenocarcinoma (n = 73), defined by International Classifications of Diseases for Oncology, 2^nd^ edition, codes of 80413–80493, 80702–80708, and 81403–82508, respectively. Controls were alive and cancer-free at the time of case diagnosis and matched to cases (1∶1) on age at randomization (+/−1 year) and date of baseline serum collection (+/−30 days).

### Serum 25-hydroxyvitamin D determination

Fasting serum samples were collected at baseline and stored at −70°C. 25(OH)D was measured at Heartland Assays, Inc. (Ames, IA) with the DiaSorin Liaison 25(OH)D TOTAL assay platform using a direct, competitive chemiluminescence immunoassay [8], [9]. Each batch contained matched case/control sets and blinded quality control samples, comprising 5% of the total sample number. Intrabatch and interbatch coefficients of variation were calculated using a nested components of variance analysis [10], and ranged between 9.3%–11.0% and 12.3%–13.6%, respectively.

### Statistical analysis

Case and control characteristics were compared using Wilcoxon rank sum or chi-square tests. Correlations were determined among controls using Spearman's rank order coefficient. Odds ratios (OR) and 95% confidence intervals (CI) were calculated using conditional logistic regression. To account for seasonal variation in 25(OH)D concentrations, season-specific and season-standardized quintiles of 25(OH)D were created, and models for each 25(OH)D classification were stratified on season of blood collection. The season-specific 25(OH)D quintiles were created based on month of blood collection (“darker months” = November–April and “sunnier months” = May–October). The season-standardized quintiles were created from the residuals from a model regressing log transformed 25(OH)D against calendar week of blood collection [9]. Pre-defined cutpoints for 25(OH)D, based on clinical definitions in the literature [11]–[13], were also examined. The cutpoints were <25, 25 to <37.5, 37.5 to <50, 50 to <75 (referent category), and ≥75 nmol/L. The referent category was chosen to mirror that used in other studies of circulating 25(OH)D and cancer [9], [14], [15]; it includes the mean concentration of the US population [16], and reflects sufficiency as recently defined by the Institute of Medicine [17]. This allows for calculation of risk estimates at high and low 25(OH)D concentrations compared to subjects with a “sufficient” 25(OH)D concentration. We also included risk estimates using the lowest pre-defined category as the referent. Tests for linear trend were obtained by assigning to each category an ordinal value (1–5) and treating this parameter as a continuous variable. 25(OH)D was also modeled as a continuous variable, based on a 10 nmol/L change in the original concentration and a one-residual unit change in the season-standardized classification; results using a log-transformed continuous variable were nearly identical to the latter and are not shown.

Factors tested as confounders included age, height, weight, body mass index, number of cigarettes smoked per day, years and pack-years of smoking, education, physical activity, family history of lung cancer, alcohol intake; serum β-carotene, α-tocopherol, retinol, and cholesterol; and the trial supplementation. We did not consider vitamin D intake or supplement use as these would be direct determinants of 25(OH)D. None of the aforementioned factors changed the 25(OH)D coefficients by more than 10% when added to the univariate models. However, we present a multivariate model adjusted for common lung cancer risk factors, including smoking (cigarettes/day and years smoked, both continuous), body mass index (continuous), serum cholesterol (continuous), trial supplementation (α-tocopherol yes/no, β-carotene yes/no), and alcohol intake (categorical with separate categories for non-drinkers and those with missing data).

Subgroup analyses stratified on median age, body mass index, cigarettes/day, years smoked, alcohol intake, vitamin D intake; serum α-tocopherol, β-carotene, retinol and cholesterol; leisure physical activity (moderate and heavy vs. sedentary) and trial supplementation, were conducted using unconditional logistic regression, adjusting for the matching factors. The season-specific 25(OH)D measure was used, split at quintiles 4–5 vs. quintiles 1–3. Models stratified on follow-up time (<10 years and ≥10 years), stage (1–2 vs. 3–4), and histology (small cell carcinoma, squamous cell carcinoma, and adenocarcinoma), were run conditionally. Effect modification was statistically evaluated by comparing models with and without a cross-product interaction term (25(OH)D crossed with the effect modifier) using the log-likelihood ratio test. Statistical analyses were performed using SAS software version 9.1.3 (SAS Institute, Inc., Cary, North Carolina) and all *P*-values were 2-sided.

## Results

Lung cancer cases smoked more cigarettes for a longer period of time, had lower body mass index and education, were more sedentary, and more likely to have a family history of lung cancer than controls. Cases also had lower baseline concentrations of serum β-carotene, retinol, and α-tocopherol; and higher alcohol intake compared with controls (Table 1). Serum 25(OH)D was significantly correlated with body mass index (r = 0.11); serum cholesterol (r = −0.10), retinol (r = 0.11) and α-tocopherol (r = 0.14); and intakes of vitamin D (r = 0.33), fish (r = 0.34), milk (r = −0.24), and alcohol (r = 0.15) (all p<0.02). Serum 25(OH)D was not correlated with smoking: r = −0.02, p = 0.73 for total cigarettes/day; r = −0.04, p = 0.40 for years of smoking; and r = −0.05, p = 0.28 for pack-years of smoking.

**Table 1:** Selected baseline characteristics of cases and controls,^1^ Alpha-Tocopherol, Beta-Carotene Cancer Prevention Study.

Characteristic	Cases, n = 500	Controls, n = 500	P^2^
Age (y)	59 (55–62)	59 (55–62)	matched
Body mass index (kg/m^2^)	25.4 (22.9–27.9)	25.8 (23.6–28.1)	0.02
Blood collection in darker months (Nov–April)	60.0%	58.4%	matched
Cigarettes smoked/day	20 (20–30)	20 (15–25)	<0.0001^3^
Years smoked	40 (35–44)	38 (32–43)	<0.0001
Pack-years of smoking	42 (34–54)	34 (23–45)	<0.0001
Positive family history of lung cancer^4^	13.9%	7.4%	0.01
Education (%>elementary)	16.0%	22.0%	0.02
Physical activity in leisure			0.05
Sedentary	43.0%	38.6%	
Moderate	53.0%	54.4%	
Heavy	3.8%	7.0%	
Dietary intake/day 5			
Energy (kcal)	2547 (2120–3116)	2621 (2180–2999)	0.86
Vitamin D (µg)	4.8 (3.2–6.8)	4.7 (3.4–6.7)	0.73
Calcium (mg)	1318 (1011–1674)	1333 (1019–1697)	0.91
Fish (g)	32.4 (18.1–51.4)	32.7 (19.5–51.3)	0.42
Milk (g)	24.3 (0.0–460.0)	24.3 (0.0–460.0)	0.87
Alcohol (g of ethanol)	10.7 (1.8–26.1)	8.0 (1.6–20.8)	0.04
Dietary supplement use (%yes) 6			
Vitamin D	7.8%	6.8%	0.36
Calcium	11.8%	10.4%	0.21
Serum concentrations			
25(OH)D (nmol/L)	33.6 (20.7–50.2)	35.0 (21.5–50.5)	0.33
β-Carotene (µg/L)	165 (102–249)	190 (127–304)	<0.0001
Cholesterol (mmol/L)	6.1 (5.4–6.9)	6.2 (5.5–7.0)	0.10
Retinol (µg/L)	560 (473–650)	575 (509–670)	0.002
α-Tocopherol (mg/L)	11.3 (9.6–13.3)	11.6 (10.2–13.7)	0.02

1 All values are medians and interquartile ranges unless otherwise indicated as percentages.

2 P-values are based on Chi-square tests (for categorical variables) and Wilcoxon rank sum tests (for continuous variables).

3 Median values for number of cigarettes per day are identical because 31% of subjects reported smoking a pack of cigarettes/day ( = 20), however, the overall distributions by case/control status differ, as is evident by the interquartile ranges and the p-value.

4 Family history data available for 67.6% of subjects.

5 Dietary data available for 94.2% of subjects.

6 Only 21.2% of subjects reported any supplement use.

Regardless of approach to seasonal variation in vitamin D status, serum 25(OH)D was not associated with lung cancer risk overall. For example, multivariate-adjusted risks in the highest vs. lowest quintile were 1.08 (95% CI 0.67–1.75) and 0.83 (95% CI 0.53–1.31) using the season-specific and season-standardized 25(OH)D measures, respectively (Table 2). Compared to the referent category of 50–<75 nmol/L, neither high (≥75 nmol/L) nor low (<25 nmol/L) 25(OH)D concentrations were associated with lung cancer risk (Table 2), and there was no difference in risk between the highest and lowest categories (OR = 0.91, 95% CI 0.48–1.72 for ≥75 vs. <25 nmol/L). The multivariate-adjusted risks using a continuous 25(OH)D measure were 0.98 (95% CI 0.91–1.05, p = 0.35) for a 10 nmol/L change in 25(OH)D concentration and 0.84 (95% CI 0.66–1.07, p = 0.17) for a 1-unit change in the season-standardized residual.

**Table 2:** Association between serum 25(OH)D and risk of lung cancer, presented as season-specific quintiles, season-standardized quintiles, and clinically pre-defined cutpoints, and stratified on season of blood collection.^1^

			Quintiles			
	1	2	3	4	5	P-trend
Season-Specific^2^						
Cases/Controls, N	95/101	120/100	108/100	71/100	106/99	
Crude OR^3^ (95% CI)	1.00 (reference)	1.29 (0.87–1.90)	1.16 (0.78–1.72)	0.75 (0.49–1.15)	1.16 (0.76–1.77)	0.58
Multivariate-adjusted OR^4^	1.00 (reference)	1.28 (0.83–1.99)	1.20 (0.77–1.86)	0.83 (0.52–1.35)	1.08 (0.67–1.75)	0.58
Darker season^4^	1.00 (reference)	1.28 (0.75–2.20)	0.91 (0.52–1.57)	0.58 (0.31–1.08)	0.77 (0.41–1.45)	0.05
Sunnier season^4^	1.00 (reference)	1.46 (0.64–3.34)	2.44 (1.07–5.57)	1.89 (0.80–4.44)	1.81 (0.78–4.19)	0.24
Season-standardized^5^						
Cases/Controls, N	110/100	111/100	88/100	96/100	95/100	
Crude OR^3^ (95% CI)	1.00 (reference)	0.99 (0.69–1.44)	0.79 (0.53–1.18)	0.86 (0.58–1.28)	0.85 (0.57–1.27)	0.03
Multivariate-adjusted OR^4^	1.00 (reference)	0.96 (0.64–1.45)	0.85 (0.54–1.33)	0.95 (0.61–1.48)	0.83 (0.53–1.31)	0.47
Darker season^4^	1.00 (reference)	0.90 (0.53–1.52)	0.60 (0.32–1.10)	0.68 (0.38–1.21)	0.65 (0.37–1.14)	0.07
Sunnier season^4^	1.00 (reference)	1.03 (0.51–2.10)	1.57 (0.74–3.33)	1.70 (0.78–3.73)	1.07 (0.45–2.51)	0.42
			Categories of 25(OH)D (nmol/L)			
Clinically pre-defined cutpoints	<25	25–<37.5	37.5–<50	50–<75	≥75	
Cases/Controls, N	176/151	106/120	93/98	93/102	32/29	
Crude OR^3^ (95% CI)	1.34 (0.91–1.99)	1.01 (0.68–1.50)	1.06 (0.71–1.60)	1.00 (reference)^6^	1.22 (0.68–2.20)	0.29
Multivariate-adjusted OR^4^	1.35 (0.87–2.10)	1.08 (0.70–1.69)	1.18 (0.74–1.87)	1.00 (reference)	1.23 (0.64–2.36)	0.35
Darker season^4^	1.75 (0.98–3.13)	1.26 (0.68–2.33)	0.79 (0.40–1.55)	1.00 (reference)	1.07 (0.41–2.81)	0.01
Sunnier season^4^	0.66 (0.29–1.49)	0.96 (0.46–1.97)	1.95 (0.96–3.98)	1.00 (reference)	1.25 (0.47–3.32)	0.21

Abbreviations: 25(OH)D = 25-hydroxyvitamin D; CI = confidence interval; OR = odds ratio.

1 Darker season (300 cases/300 controls) = November–April, Sunnier season (200 cases/200 controls) = May–October.

2 Cutpoints for the season-specific quintiles were Q1: ≤16.8, Q2: >16.8 and ≤25.7, Q3: >25.7 and ≤37.1, Q4: >37.1 and ≤50.1, Q5: >50.1 nmol/L for the darker season and Q1: ≤24.3, Q2: >24.3 and ≤35.0, Q3: >35.0 and ≤46.7, Q4: >46.7 and ≤57.8, Q5: >57.8 nmol/L for the sunnier season.

3 Crude odds ratios are based on conditional logistic regression.

4 Multivariate-adjusted odds ratios are conditioned on the matching factors and adjusted for smoking (#cigarettes/day and # years smoked), body mass index, serum cholesterol, study supplementation group, and alcohol intake.

5 Cutpoints for the season-standardized quintiles were Q1: ≤3.01, Q2: >3.01 and ≤3.38, Q3: >3.38 and ≤3.65, Q4: >3.65 and ≤3.94, Q5: >3.95 residual units.

6 The referent group for the clinically pre-defined cutpoints is 50–<75 nmol/L. Data using the lowest category as the referent are reported in the text.

In contrast to the overall findings, we observed inverse associations in men whose blood was collected during the darker season of the year. Trend test p-values were 0.05 (season-specific measure), 0.07 (season-standardized measure) and 0.01 (pre-determined cutpoints)(Table 2). The individual category risk estimates and interaction tests were not statistically significant, however. The odds ratios comparing the highest (≥75 nmol/L) vs. lowest (<25 nmol/L) pre-defined vitamin D categories by season were 0.61 (95% CI 0.24–1.52) and 1.90 (95% CI 0.67–5.42) for darker and sunnier seasons, respectively. The inverse pattern in the darker season was also supported by the continuous 25(OH)D measure where multivariate-adjusted risks were 0.89 (95% CI 0.81–0.98, p = 0.02) for the darker season and 1.07 (95% CI 0.95–1.21, p = 0.25) for the sunnier season, for a 10 nmol/L change in 25(OH)D concentration, p-interaction 0.01, and 0.67 (95% CI 0.50–0.90, p = 0.01) for the darker season and 1.36 (95% CI 0.82–2.27, p = 0.23) for the sunnier season, for a 1-unit change in the season-standardized residual, p-interaction 0.04.

Analysis of selected subgroups (Table S1) showed lower risks for participants with higher serum 25(OH)D who had above median dietary and total vitamin D intake, alcohol intake, serum α-tocopherol, or body mass index, and those with below median serum β-carotene. Suggested risk elevations were noted for subjects in the placebo group of the trial supplementation (compared with the other supplementation groups), and those with small cell lung cancer (compared with squamous cell carcinoma or adenocarcinoma). None of the interactions were statistically significant, however, and no differences were noted in subgroups of disease stage, follow-up time, smoking characteristics, physical activity, or the other factors tested.

## Discussion

Circulating 25(OH)D was not associated with lung cancer risk overall in this study; however, inverse associations were suggested for subjects whose blood was collected in the darker months. The patterns were similar using pre-defined, season-specific, and season-standardized classifications of 25(OH)D and were supported by risk estimates when 25(OH)D was modeled as a continuous measure.

In addition to food and supplemental sources, vitamin D is synthesized in the skin when exposed to ultraviolet B radiation from sunlight, and hydroxylated in the liver to form 25(OH)D [1]. An additional hydroxylation in the kidney and other organs [1], [18] converts 25(OH)D to the active form 1,25-dihydroxyvitamin D [1,25(OH)_2_D] via the 1-α-hydroxylase enzyme, which is also expressed in the lung [18], [19]. Potential anti-carcinogenic activity of 1,25(OH)_2_D includes the promotion of cellular differentiation and apoptosis, and the inhibition of cellular proliferation and angiogenesis [1].

Ecologic studies have linked sunlight exposure with reduced incidence and mortality of cancer at many sites, including lung cancer [20], [21] and lung cancer survival was better for patients diagnosed in the summer or fall compared with the winter [22], [23]. The underlying biology for these observations has been attributed to vitamin D [20], [22], [23]. In a study of early-stage non-small cell lung cancer patients, higher circulating 25(OH)D [24], specific vitamin D receptor (VDR) genetic variants [25], and high vitamin D intakes combined with surgery in the summer [26] were associated with improved survival. Some VDR variants, but not circulating 25(OH)D, were associated with improved survival in advanced non-small cell lung cancer patients [27]. However, in an updated analysis of the NHANES III Study, higher 25(OH)D was associated with an increased risk of lung cancer mortality among men (RR = 1.87, 95% CI 1.04–3.34, p-trend = 0.03) [4], and in the only other prospective study of circulating vitamin D and lung cancer risk, which reported 25(OH)D values similar to those in the current study, no overall association was observed based on 122 cases [5]. The latter cohort included mainly non-smokers (58% of men and 81% of women), with no reported effect modification by smoking status or season of blood collection, but an inverse association was observed between 25(OH)D and lung cancer risk in women and subjects younger than 50 years [5].

While some evidence suggests a possible inverse association between 25(OH)D and colorectal cancer risk, the data for breast and prostate cancers are less conclusive [1], [2]. In addition, a recent pooling project of over 5,000 cancer cases suggested no benefit of higher circulating 25(OH)D for risk of non-Hodgkin lymphoma, or endometrial, ovarian, upper gastrointestinal, or kidney cancers, and possible harm for pancreatic cancer [28]–[33]. By contrast, higher serum 25(OH)D concentrations were associated with reduced risk of bladder cancer in the ATBC Study [34], suggesting that it is possible to detect cancer associations within the range of 25(OH)D concentrations observed in ATBC. The current study is one of only a few to examine 25(OH)D and lung cancer risk.

We used several approaches to account for seasonal variation in 25(OH)D concentrations because a simulation study indicated that results could be biased either toward or away from the null if seasonal variation was not properly considered [35]. We selected an equal number of cases based on month of blood collection, with controls matched within 30 days. Analyses were conducted using season-specific and season-standardized 25(OH)D quintiles, and were stratified on season, the latter of which indicated that 25(OH)D may be inversely associated with lung cancer risk among subjects with blood collected during the darker months. Circulating 25(OH)D is considered to best reflect vitamin D status when measured in the winter [1], and in a twin study, genetic factors explained variability of 25(OH)D status only when measured in the winter [36]. Therefore, the inverse association we noted during the darker months could reflect the use of a more accurate measure of usual, chronic, vitamin D status that is not influenced by episodic sun exposure in summer. Alternatively, maintaining higher vitamin D status during the longer period of winter months in higher latitudes may be biologically important for the vitamin D-cancer association; Vieth has hypothesized that sharp declines in serum 25(OH)D in the winter and a lag in the compensating response of cellular hydroxylases involved in vitamin D metabolism (e.g., CYP24 and CYP27B1) could be detrimental in terms of cancer risk [37]. This association could also simply be due to chance and should be examined in other studies.

The prospective design of the current study, with up to 20 years of follow-up, reduces any potential for an effect of cancer on the 25(OH)D concentrations. All participants were current smokers at baseline, but as no apparent confounding by smoking duration or intensity was observed, and 25(OH)D was not correlated with any of our measures of smoking, any effects due to residual confounding from smoking are expected to be minimal. Given that smoking is such a strong risk factor for lung cancer, however, it is important to define additional lung cancer risk factors in the context of smoking. Circulating 25(OH)D incorporates the contributions of dietary and supplemental vitamin D intake, as well as vitamin D due to sun exposure, and is considered the accepted biomarker of vitamin D status [1]. Thus, 25(OH)D more accurately represents vitamin D status than proxy measures such as latitude, sun exposure, dietary intake, or season of diagnosis.

Vitamin D status in the study population was generally quite low (median = 35.0 nmol/L among controls) due to limited vitamin D synthesis that occurs at high latitudes in the winter months [1], few study blood collections in the peak summer months, and because vitamin D supplement use was uncommon in this population. It is unclear whether inverse associations would be more evident at higher 25(OH)D concentrations. Only one measurement of 25(OH)D was used to represent individual long-term vitamin D exposure; however, 25(OH)D has been correlated in samples collected from the same individuals 3–14 years apart [38]–[40]. Since our study subjects were 50–69 year old men, we were unable to re-examine the previous findings of an inverse association among women and subjects younger than 50 years [5] and our findings may not be generalizable to younger, female, and/or non-smoking populations.

In summary, in the overall analysis of this prospective study, 25(OH)D was not associated with lung cancer risk using the season-specific, season-standardized, or pre-defined classifications of 25(OH)D. However, in analyses stratified on season of blood collection, an inverse association between 25(OH)D status and lung cancer risk was suggested when blood was collected during the darker months of the year, a time when skin synthesis of 25(OH)D is reduced. Future studies of vitamin D status and lung cancer risk should specifically examine associations stratified by season of blood collection.

## Supporting Information

Table S1 **Association between serum 25(OH)D and risk of lung cancer, presented as season-specific quintiles, stratified by selected baseline and clinical characteristics**
(DOC)
