# Supplementary material for: Serum 25-Hydroxyvitamin D and Risk of Lung Cancer in Male Smokers: A Nested Case-Control Study
Source: PLoS One. 2011 Jun 10;6(6):e20796. doi: 10.1371/journal.pone.0020796 (PMC3112221; doi:10.1371/journal.pone.0020796)
Supplement: Table S1 — Association between serum 25(OH)D and risk of lung cancer, presented as season-specific quintiles, stratified by selected baseline and clinical characteristics (DOC) [file pone.0020796.s001.doc]

**Table S1**. Association between serum 25(OH)D and risk of lung cancer, presented as season-specific quintiles, stratified by selected baseline and clinical characteristics

| Stratification |  | Quintiles 1-3 | Quintiles 4-5 |
| --- | --- | --- | --- |
| Age (years) |  | | |
| < 59 | Cases/Controls, N | 157/153 | 83/92 |
|  | OR^1^ (95% CI) | 1.00 (reference) | 0.88 (0.61-1.28) |
| > 59 | Cases/Controls, N | 166/148 | 94/107 |
|  | OR^1^ (95% CI) | 1.00 (reference) | 0.78 (0.55-1.12) |
| Body mass index (kg/m^2^) | | | |
| < 25.8 | Cases/Controls, N | 181/160 | 100/90 |
|  | OR^1^ (95% CI) | 1.00 (reference) | 0.98 (0.69-1.40) |
| > 25.8 | Cases/Controls, N | 142/141 | 76/109 |
|  | OR^1^ (95% CI) | 1.00 (reference) | 0.69 (0.47-1.01) |
| # Cigarettes/day |  |  |  |
| < 20 | Cases/Controls, N | 68/124 | 56/94 |
|  | OR^1^ (95% CI) | 1.00 (reference) | 1.05 (0.67-1.64) |
| > 20 | Cases/Controls, N | 255/177 | 121/105 |
|  | OR^1^ (95% CI) | 1.00 (reference) | 0.80 (0.58-1.11) |
| Years of smoking |  |  |  |
| < 38.5 | Cases/Controls, N | 129/154 | 63/96 |
|  | OR^1^ (95% CI) | 1.00 (reference) | 0.79 (0.53-1.18) |
| > 38.5 | Cases/Controls, N | 194/147 | 114/103 |
|  | OR^1^ (95% CI) | 1.00 (reference) | 0.85 (0.60-1.20) |
| Alcohol intake (g/day)^2^ | |  |  |
| < 8.0 | Cases/Controls, N | 138/153 | 70/82 |
|  | OR^1^ (95% CI) | 1.00 (reference) | 0.93 (0.63-1.38) |
| > 8.0 | Cases/Controls, N | 165/135 | 92/107 |
|  | OR^1^ (95% CI) | 1.00 (reference) | 0.71 (0.49-1.01) |
| Dietary vitamin D (g/day) ^2^ | | |  |
| < 4.75 | Cases/Controls, N | 170/175 | 61/62 |
|  | OR^1^ (95% CI) | 1.00 (reference) | 1.02 (0.67-1.54) |
| > 4.75 | Cases/Controls, N | 133/113 | 101/127 |
|  | OR^1^ (95% CI) | 1.00 (reference) | 0.68 (0.47-0.97) |
| Total vitamin D (g/day) ^2^ | | |  |
| < 4.94 | Cases/Controls, N | 164/177 | 53/62 |
|  | OR^1^ (95% CI) | 1.00 (reference) | 0.93 (0.61-1.43) |
| >4.94 | Cases/Controls, N | 139/111 | 109/127 |
|  | OR^1^ (95% CI) | 1.00 (reference) | 0.67 (0.47-0.97) |
| Serum α-tocopherol (mg/L) | | |  |
| < 11.6 | Cases/Controls, N | 178/164 | 90/85 |
|  | OR^1^ (95% CI) | 1.00 (reference) | 0.97 (0.68-1.40) |
| > 11.6 | Cases/Controls, N | 145/137 | 87/114 |
|  | OR^1^ (95% CI) | 1.00 (reference) | 0.71 (0.49-1.03) |
| Serum β-carotene (ug/L) | | |  |
| < 190 | Cases/Controls, N | 205/154 | 88/95 |
|  | OR^1^ (95% CI) | 1.00 (reference) | 0.69 (0.48-0.99) |
| > 190 | Cases/Controls, N | 118/147 | 89/104 |
|  | OR^1^ (95% CI) | 1.00 (reference) | 1.06 (0.73-1.54) |
| Serum retinol (ug/L) |  |  |  |
| < 575 | Cases/Controls, N | 179/159 | 88/89 |
|  | OR^1^ (95% CI) | 1.00 (reference) | 0.88 (0.61-1.27) |
| > 575 | Cases/Controls, N | 144/142 | 89/110 |
|  | OR^1^ (95% CI) | 1.00 (reference) | 0.80 (0.55-1.15) |
| Serum cholesterol (mmol/L) | | |  |
| < 6.22 | Cases/Controls, N | 172/146 | 103/105 |
|  | OR^1^ (95% CI) | 1.00 (reference) | 0.83 (0.59-1.18) |
| > 6.22 | Cases/Controls, N | 151/155 | 73/94 |
|  | OR^1^ (95% CI) | 1.00 (reference) | 0.82 (0.56-1.20) |
| Physical activity |  |  |  |
| Sedentary | Cases/Controls, N | 154/138 | 61/55 |
|  | OR^1^ (95% CI) | 1.00 (reference) | 1.00 (0.65-1.53) |
| Moderate/heavy | Cases/Controls, N | 168/163 | 116/144 |
|  | OR^1^ (95% CI) | 1.00 (reference) | 0.77 (0.56-1.07) |
| Trial supplementation | |  |  |
| Placebo | Cases/Controls, N | 74/81 | 43/36 |
|  | OR^1^ (95% CI) | 1.00 (reference) | 1.34 (0.78-2.32) |
| β-Carotene only | Cases/Controls, N | 78/68 | 49/55 |
|  | OR^1^ (95% CI) | 1.00 (reference) | 0.76 (0.46-1.27) |
| α-Tocopherol only | Cases/Controls, N | 80/80 | 35/52 |
|  | OR^1^ (95% CI) | 1.00 (reference) | 0.69 (0.40-1.17) |
| β-Carotene and  α-tocopherol | Cases/Controls, N | 91/72 | 50/56 |
|  | OR^1^ (95% CI) | 1.00 (reference) | 0.72 (0.44-1.18) |
| Follow-up period (years) |  |  |  |
| < 10 | Cases/Controls, N | 175/160 | 85/100 |
|  | OR^3^ (95% CI) | 1.00 (reference) | 0.75 (0.51-1.10) |
| > 10 | Cases/Controls, N | 148/141 | 92/99 |
|  | OR^3^ (95% CI) | 1.00 (reference) | 0.89 (0.61-1.28) |
| Case stage^4^ |  |  |  |
| Stage 1-2 | Cases/Controls, N | 63/65 | 29/27 |
|  | OR^3^ (95% CI) | 1.00 (reference) | 1.13 (0.57-2.27) |
| Stage 3-4 | Cases/Controls, N | 142/126 | 82/98 |
|  | OR^3^ (95% CI) | 1.00 (reference) | 0.73 (0.49-1.08) |
| Histology^5^ |  |  |  |
| Small cell carcinoma | Cases/Controls, N | 57/63 | 43/37 |
|  | OR^3^ (95% CI) | 1.00 (reference) | 1.33 (0.72-2.46) |
| Squamous cell carcinoma | Cases/Controls, N | 127/110 | 52/69 |
|  | OR^3^ (95% CI) | 1.00 (reference) | 0.65 (0.42-1.02) |
| Adenocarcinoma | Cases/Controls, N | 47/41 | 25/31 |
|  | OR^3^ (95% CI) | 1.00 (reference) | 0.68 (0.34-1.39) |

^1^Odds ratios are based on unconditional logistic regression, adjusted for the matching factors.

^2^Dietary data available for 94.2% of subjects.

^3^ Odds ratios are based on conditional logistic regression.

^4^ Stage data available for 63% of cases.

^5^ Histology data available for 70% of cases.
